# Picomolar Detection of Lead Ions (Pb^2+^) by Functionally Modified Fluorescent Carbon Quantum Dots from Watermelon Juice and Their Imaging in Cancer Cells

**DOI:** 10.3390/jimaging9010019

**Published:** 2023-01-16

**Authors:** Kundan Singh Rawat, Vikram Singh, Chandra Prakash Sharma, Akanksha Vyas, Priyanka Pandey, Jagriti Singh, Neeraj Mohan Gupta, Monika Sachdev, Atul Goel

**Affiliations:** 1 Fluorescent Chemistry Lab, Division of Medicinal and Process Chemistry, CSIR-Central Drug Research Institute, Lucknow 226031, India; 2 Academy of Scientific and Innovative Research (AcSIR), Ghaziabad 201002, India; 3 Division of Endocrinology, CSIR-Central Drug Research Institute, Lucknow 226031, India

**Keywords:** carbon quantum dots, watermelon juice, green synthesis, lead ion sensing, bioimaging

## Abstract

Water contamination due to the presence of lead is one of the leading causes of environmental and health hazards because of poor soil and groundwater waste management. Herein we report the synthesis of functionally modified luminescent carbon quantum dots (CQDs) obtained from watermelon juice as potential nanomaterials for the detection of toxic Pb^2+^ ions in polluted water and cancer cells. By introducing surface passivating ligands such as ethanolamine (EA) and ethylenediamine (ED) in watermelon juice, watermelon-ethanolamine (WMEA)-CQDs and watermelon-ethylenediamine (WMED)-CQDs exhibited a remarkable ~10-fold and ~6-fold increase in fluorescence intensity with respect to non-doped WM-CQDs. The relative fluorescence quantum yields of WMEA-CQDs and WMED-CQDs were found to be 8% and 7%, respectively, in an aqueous medium. Among various functionally-modified CQDs, only WMED-CQDs showed high selectivity towards Pb^2+^ ions with a remarkably good limit of detection (LoD) of 190 pM, which is less than that of the permissible limit (72 nM) in drinking water. The functionally altered WMED-CQDs detected Pb^2+^ metal ions in polluted water and in a human cervical cancer cell line (HeLa), thus advocating new vistas for eco-friendly nanomaterials for their use as diagnostic tools in the environment and biomedical research areas.

## 1. Introduction

Lead is one of the most abundant and toxic substances in the category of heavy metals [1]. It is found in the environment due to its presence in many anthropogenic sources, such as electronic waste, combustion of leaded gasoline, and unregulated industrial emissions [2]. Since lead is harmful to the environment and accumulates in soil and groundwater, it is directly linked to human health [3]. Even a very low concentration of toxic lead ions exposure can cause reproductive, neurological, developmental, and heart disorders in humans [4]. Considering the deposition of lead in the human body, the U.S. Environmental Protection Agency (EPA) has set the permissible limit of lead in drinking water at 15 ppb (<72 nM) [5]. Once introduced into the body, lead can induce serious health problems, such as hypertension, dysgenesis, memory loss, anaemia, mental confusion, and reduced IQ level in children [6]. In April 2014, there was a water crisis in Flint city (Michigan, USA) due to the leakage of Pb^2+^ ions from ageing pipes into flint river, which affected more than 1 lac residents [7]. Therefore, developing an efficient, rapid and cost-effective method for the detection of lead is crucial in monitoring lead poisoning in the environment [8]. The common protocols for detection of heavy metal ions include anodic stripping voltammetry [9], atomic absorption spectrometry [10], and inductively coupled plasma mass spectrometry [11]. These techniques can measure the total lead content [12] and often require extensive sample preparation and technical knowledge of the equipment. Thus, a simple and inexpensive method is needed to detect lead ions in environmental, biological, and industrial samples. The fluorometric method has various advantages, such as high sensitivity, low cost and simplicity for detecting metal ions. Numerous fluorescent organic dyes for the detection of Pb^2+^ ions have been developed in the last few decades [13,14]. However, there are some limitations associated with organic dyes, such as relatively complicated synthesis and purification, fixed excitation-based emission, and photo-bleaching. 

In recent years, carbon quantum dots (CQDs) derived from natural sources including beetroot [15,16], watermelon [17,18], orange juice [19], banana juice [20], strawberries [21], garlic [22], potatoes [23], and some more environmentally benign sources [24,25,26,27,28], have become an area of growing interest due to their green eco-friendly simple one-pot preparation, remarkable excitation-dependent emission, excellent photo-stability, high biocompatibility, and aqueous dispersibility [29]. Numerous fluorescent CQDs are reported in the literature for the detection of biometal ions such as Zn, Fe, Cu, and Co metal ions [30,31,32,33,34,35,36,37]; however, few reports are about Pb^2+^ ions detection [38,39,40,41,42,43,44,45,46,47,48,49,50,51,52] in which most of them generally display fluorescence quenching phenomena upon binding with Pb^2+^ ions (Supplementary Table S1).

## 2. Materials and Methods

### 2.1. Synthesis of CQDs from Watermelon Juice

**Scheme 1:**
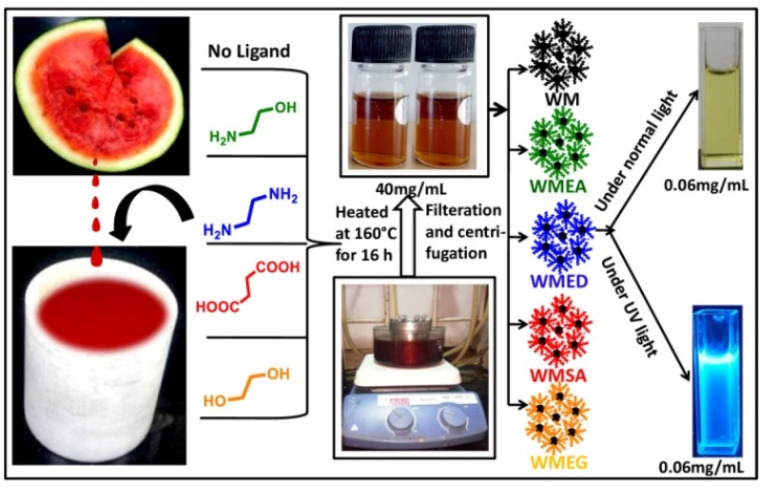
Graphical representation of the one-pot synthesis of CQDs from watermelon juice by adding different surface passivating agents.

Briefly, a juicer, watermelon juice was taken out, and 20 mL of juice was filtered using a Whatman filter paper Grade-I (11 microns); the filtered juice was poured into a Teflon-coated autoclave (50 mL) and the mixture was heated at 160 °C for 16 h as shown in Scheme 1. Subsequently, the resultant CQDs mixture was purified by filtration through a 0.2 µm syringe filter and repeated centrifugation at 8000 rpm for 20 min. A clear brownish solution of CQDs was obtained, which was termed as WM-CQDs. The same procedure was followed for preparing CQDs with surface passivating ligands (2 gm in 20mL watermelon juice) such as ethanol amine (WMEA), ethylene diamine (WMED), succinic acid (WMSA), and ethylene glycol (WMEG).

### 2.2. General Instrument Information

UV-visible electronic absorption spectra measurements were carried out using an Agilent Cary 100-UV-visible Spectrophotometer. The fluorescence emission spectra of the samples were measured using a Horiba Fluoromax 4C spectrofluorometer (HORIBA, Edison, NJ, USA). Fluorescence lifetime experiments were performed using a Horiba Jobin Yvon instrument (HORIBA, Edison, NJ, USA) in a Time-Correlated Single Photon Counting (TCSPC). A nano-LED of 390 nm was used as a source of excitation for CQDs. LUDOX AM30 (Sigma-Aldrich/ St. Louis, MO, USA) colloidal silica scattering medium was used for the instrument response function. The pulse repetition rate of TCSPC was fixed at 1 MHz. The average size of CQDs was carried out by using an FEI Titan G2 (Hillsboro, OR, USA) 300 kV high-resolution transmission electron microscope (HR-TEM). Surface functionality characterization was carried out by X-ray photoelectron spectroscopy (XPS) using PHI 5000 Versaprobe II (ULVAC-PHI, Enzo Chigasaki, Japan), FEI.

### 2.3. Culture and Maintenance of HeLa Cells

Human cervical cancer cell line (HeLa) was cultured in Dulbecco’s Modified Eagles Medium (DMEM) with L-glutamine, phenol red, and D-glucose (4.8 g, Sigma, St. Louis, MO, USA) supplemented with 10% heat-inactivated Fetal Bovine Serum (FBS) (Gibco, New York, NY, USA) and 1% Anti-Anti, i.e., antibiotic cocktail (Gibco). These cells were analyzed for their proper density and morphology at 37 °C in a CO_2_ incubator. After the cells were 70–80% confluent, these cells were trypsinised and seeded (~3 × 10^4^) onto an 18mm round cover-slip inside the 12-well plate and incubated overnight with complete media (approximately 12 h) in 37 °C in a CO_2_ incubator. The next day, culture media were replaced with fresh culture media containing Pb^2+^ (lead) in three concentrations of 0.1 μM, 1 μM, and 10 μM and incubated for 30 min in a 37 °C in a CO_2_ incubator. Now, these HeLa cells were gently washed with 1× PBS (Phosphate-Buffered Saline) twice, and then WMED (100 μL of 0.06 mg/mL or 0.128 mg/mL) was incubated for 1 h in a 37 °C in a CO_2_ incubator. Then, HeLa cells were gently washed with 1× PBS twice, followed by fixing with 4% PFA (Paraformaldehyde) for 10 min at room temperature. Fixed cells were rewashed with 1× PBS twice; then, nuclear staining was carried out with dye TO-PRO (Invitrogen) at a concentration of 1 µM for 20 min at room temperature; finally, these cells were washed twice with 1× PBS. Then, coverslips were mounted upside down with Prolong gold antifade reagent (Invitrogen) on clean glass slides and sealed with nail enamel.

### 2.4. Confocal Microscopy

Confocal microscopy was performed using a confocal microscope LEICA TCS SP8 (LEICA, Mannheim, Germany). Different cells were analyzed, and images were captured for multiple segments with an excitation of 405 nm for WMED-CQDs while the emissions were collected within a band pass of 450–510 nm. Bright-field images were also captured to identify specific fields of examination of the HeLa cells.

## 3. Results and Discussion

This watermelon (Citrullus lanatus) juice contains a considerable amount of citrulline (amino acid), lycopene (carotenoid), and different phenolic compounds. The pink colour of watermelon is due to the presence of lycopene, a bright red carotenoid hydrocarbon. We demonstrated a one-pot green method for the synthesis of fluorescent CQDs from watermelon juice in the absence and presence of different surface passivating ligands such as (Ethanol amine (EA), Ethylene diamine (ED), Succinic acid (SA), and Ethylene glycol (EG)) using hydrothermal methodology as shown in Scheme 1.

**Figure 1:**
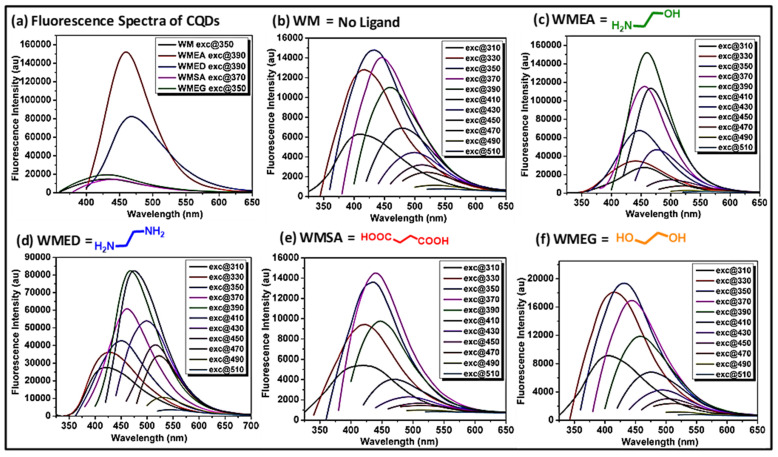
(**a**) Emission spectra of WM-CQDs and WMEG at 350 nm excitation, emission spectra of WMEG-CQDs at 370 nm excitation, emission spectra of WMEA-CQDs and WMED-CQDs at 390 nm excitation, showing intensity difference by introducing different surface passivating agents. Excitation-dependent emission spectra of (**b**) WM-CQDs; (**c**) WMEA-CQDs; (**d**) WMED-CQDs; (**e**) WMSA-CQDs; and (**f**) WMEG-CQDs obtained by hydrothermal treatment at different excitation.

The excitation-dependent emission properties of synthesized CQDs are shown in Figure 1 and Supplementary Figure S1. First, we collected the emission of carbon quantum dots upon their excitation (where fluorescence intensity was maximum) and found that the fluorescence intensity was increased by 10-fold and 6-fold in the case of WMEA and WMED, respectively, as compared to CQDs without being ligand-treated (Figure 1a). In other cases (WMEG, WMSA), no significant change in the fluorescence intensity was observed. Then, we checked the excitation-dependent emission behavior of all the synthesized carbon quantum dots as shown in Figure 1b–f. Based on its fluorescence intensity, we selected WMEA and WMED for detailed studies. The solution of ethanolamine ligand-treated watermelon CQDs (WMEA) firmly absorbed at 250–400 nm with absorbance maxima at 305 nm due to π–π* transition (Supplementary Figure S1a and Supplementary Table S2, black). WMEA-CQDs exhibited blue fluorescence with an emission maximum at 468 nm on excitation at 390 nm wavelength (Supplementary Figure S1a, blue). The solution of WMED-CQDs dynamically absorbed at 250–410 nm with absorbance maxima at 315 nm due to π–π* transition (Figure S1b, black). WMED-CQDs also exhibited blue fluorescence with an emission maximum at 470 nm on excitation at 390 nm wavelength (Supplementary Figure S1b, blue). To explore excitation-dependent emission property of these CQDs, i.e., without and with ligand treated, we examined the sample with excitation from 310 to 510 nm having 20 nm interval and plotted their emission spectra (Figure 1a–e). A good red shift in all the cases was observed with maximum intensity at 350 nm in the case of WM and WMSA, 390 nm in the case of WMEA, and WMED and 370 nm in the case of WMEG (Figure 1f). By incorporating the surface passivating ligands in watermelon juice, a remarkable ~10-fold increase in fluorescence intensity was observed in the case of WMEA-CQDs, and a ~6-fold enhancement in fluorescent intensity was obtained in the case of WMED-CQDs. In the case of WMSA and WMEG CQDs, no significant change in intensity was observed. The fluorescence enhancement may originate due to incorporation of nitrogen-containing ligands (EA, ED) to form poly-heteroaromatic structures and surface functionalized with nitrogen atoms on carbon dots. Literature reports also revealed that nitrogen doping on CQDs are useful to make them stable colloidal solution, resulting in enhancement of fluorescence intensity.

In an aqueous medium taking fluorescein dye as a standard reference, the relative quantum yields of both CQDs, i.e., WMEA-CQDs and WMED-CQDs were found to be 8% and 7%, respectively. In order to check the photo- and shelf-storage stability of CQDs as reported in the literature [53], both the CQDs were irradiated by continuous UV light exposure for up to ~8 h, which revealed that these CQDs were stable as no significant change in fluorescence intensity was observed (Supplementary Figure S2). These CQDs were found to be stable after 12 months of storage in a domestic refrigerator. In order to study the pH-dependent stability of these CQDs, different pH (1–12) solutions were prepared for analysis. WMEA-CQDs showed almost stable fluorescence between pH 4–12, while WMED-CQDs showed stability between pH 1–10 (Supplementary Figure S3).

**Figure 2:**
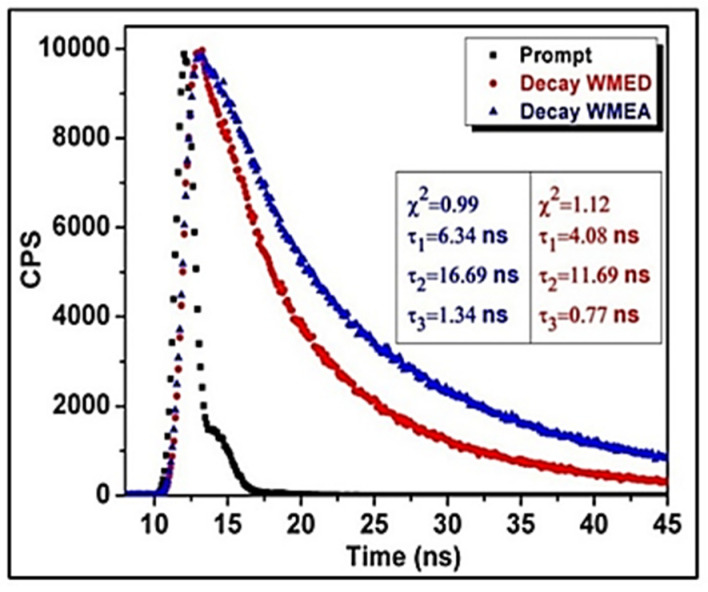
Time-resolved fluorescence decay of WMEA-CQDs (λ_ex_ = 390 nm and λ_em_ = 460 nm) and WMED-CQDs (λ_ex_ = 390 nm and λ_em_ = 470 nm) in aqueous medium.

Time-correlated single-photon counting (TCSPC) experiment was carried out to measure the average fluorescence lifetime of both the CQDs, i.e., WMEA-CQDs and WMED-CQDs, taking Ludox as a prompt. The results revealed that both the CQDs fitted in tri-exponential function, and the lifetime decay of WMEA-CQDs and WMED-CQDs is shown in Figure 2. The average fluorescence was found to be 15.34 ns and 10.85 ns for WMEA-CQDs and WMED-CQDs, respectively (Figure 2).

**Figure 3:**
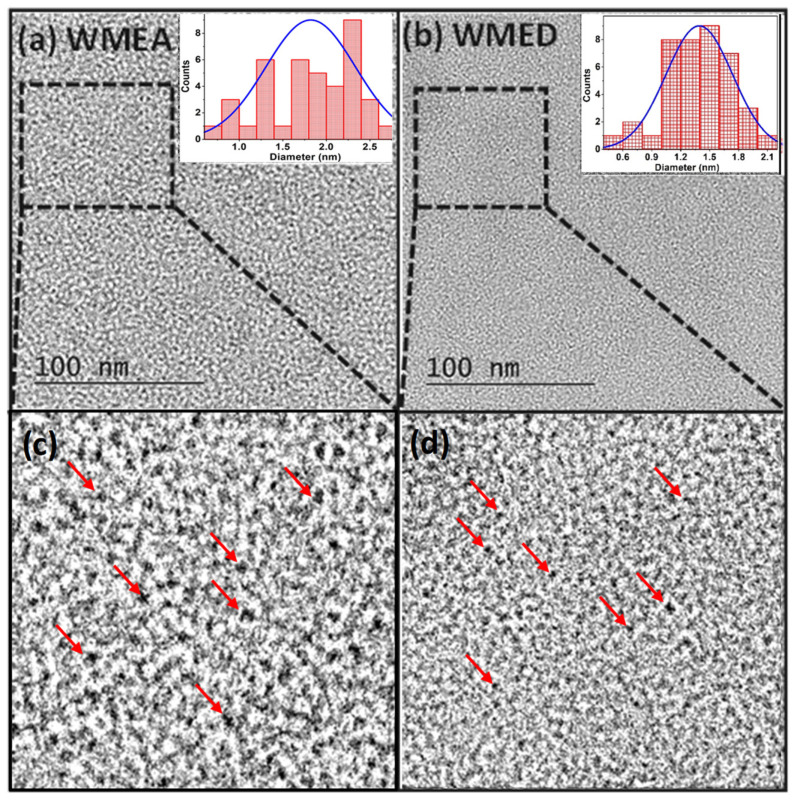
TEM images of watermelon-CQDs (**a**) WMEA CQDs dispersed in water; inset shows particle size distribution and (**b**) WMED CQDs dispersed in water; inset shows particle size distribution; (**c**,**d**) zoomed TEM images of CQDs, red arrows showed CQDs.

To determine the morphology and core sizes of water-miscible CQDs, i.e., WMEA-CQDs and WMED-CQDs from hydrothermal treatment, a High-Resolution Transmission Electron Microscope (HR-TEM) analysis was performed. Both the CQDs (WMEA and WMED) showed spherical morphology (Figure 3), having average particle sizes of 1.8 nm and 1.4 nm for WMEA and WMED, respectively (Figure 3a,b inset).

**Figure 4:**
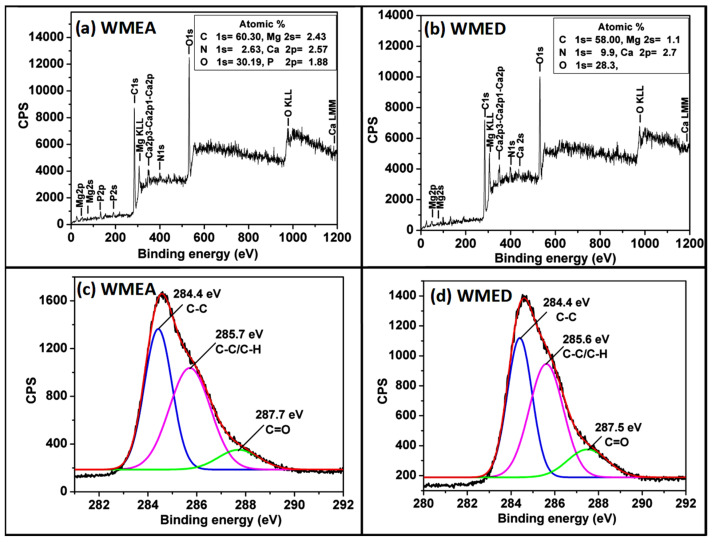
Full range XPS analysis of watermelon-CQDs prepared by adding (**a**) ethanol amine; (**b**) ethylene diamine; high-resolution XPS spectra of the C1s region of watermelon-CQDs synthesized by adding (**c**) ethanol amine; (**d**) ethylene diamine.

Fourier-transform infrared spectroscopy (FT-IR) and X-ray photoelectron spectroscopy (XPS) analyses were carried out to determine the surface functionalities of the synthesized CQDs. Figure 4a showed the full XPS spectrum of the WMEA with eleven peaks at 46, 75, 134, 187, 285, 307,348, 398, 530, 978, and 1187 eV attributed to Mg2p, Mg2s, P2p, P2s, C1s, Mg KLL, Ca2p3-Ca2p1-Ca2p, N1s, O1s, O KLL, and Ca LMM, respectively. Binding energy values were measured from the high-resolution spectrum of C1s for WMEA CQDs (Figure 4c, for O1s, N1s: Supplementary Figure S4a,b) showed three different chemical environments, i.e., C=C at 284.4 eV, C-C/C-H at 285.7 eV, and C=O at 287.7 eV. Similarly, Figure 4b showed the full XPS spectrum of the WMED with ten peaks at 49, 72, 285, 307, 348, 397, 437, 530, 977, and 1184 eV attributed to Mg2p, Mg2s, C1s, Mg KLL, Ca2p3-Ca2p1-Ca2p, N1s, Ca2s, O1s, O KLL, and Ca LMM, respectively.

Binding energy obtained from a high-resolution spectrum of C1s for WMED (Figure 4d, for O1s, N1s: Supplementary Figure S4c,d) also showed carbon has three different chemical environments, i.e., C=C at 284.4 eV, C-C/C-H at 285.6 eV, and C=O at 287.5 eV. The data revealed the composition of both the CQDs according to which C, N, and O were present in higher amounts, and a trace amount of Mg, Ca, and P were also present.

In addition, functional groups on the surface of CQDs (for ethanolamine-treated and ethylene diamine treated) were also characterized from FTIR spectra. Ethanolamine-treated CQDs (WMEA) showed peaks at 3744 for 3354 cm^−1^ for stretching vibrations of C-OH, 3745 cm^−1^ for O-H stretching for primary alcohol, 3649 cm^−1^ and 3592 cm^−1^ for N-H stretching for primary amine, 2855 and 2924 cm^−1^ for C-H stretching, 2364 cm^−1^ for O=C-O stretching, 1702 cm^−1^ for C=O stretching, 1648 cm^−1^ for C=O stretching of amide, 1543 and 1515 cm^−1^ for N-O stretching, 1461 cm^−1^ for aromatic C=C stretching and 1394 cm^−1^ for C-H bending vibrations. Ethylene diamine treated CQDs (WMED) exhibited peaks at 3352 cm^−1^ for stretching vibrations of C-OH, 3744 cm^−1^ for O-H stretching for primary alcohol, 3644 cm^−1^ and 3536 cm^−1^ for N-H stretching of amine, 2927 cm^−1^ for C-H stretching, 2362 cm^−1^ for O=C-O stretching, 1646 cm^−1^ for C=O stretching of amide, 1425 cm^−1^ for aromatic C=C stretching, 1392 cm^−1^ for C-H bending vibrations. FTIR and XPS studies indicated that watermelon-CQDs are functionalized with hydroxyl, carbonyl, carboxylic acid, and amide groups.

**Figure 5:**
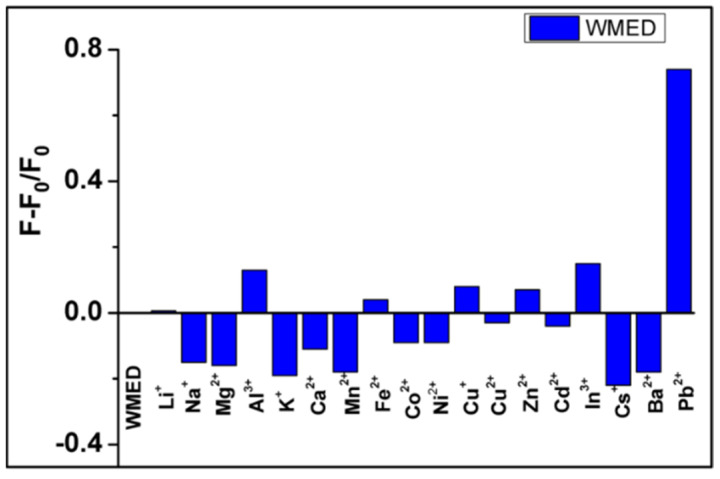
Selectivity of the WMED to different metal ions. The concentration of Pb^2+^ and other metal ions was 1 × 10^−4^ M.

Perchlorate salts of various metal cations were used to conduct the selectivity studies for both CQDs, i.e., WMEA and WMED (Figure 5, Supplementary Figure S5). These experiments showed that only lead (Pb^2+^) triggered a dramatic increase in emission intensity with maxima at 468 nm. In contrast, as shown in Figure 5, other cations were either quenched or showed minor effects in the case of WMED-CQDs (Supplementary Figure S5) and, for WMEA CQDs, no selectivity for metal ions was observed. (Supplementary Figure S6).

**Figure 6:**
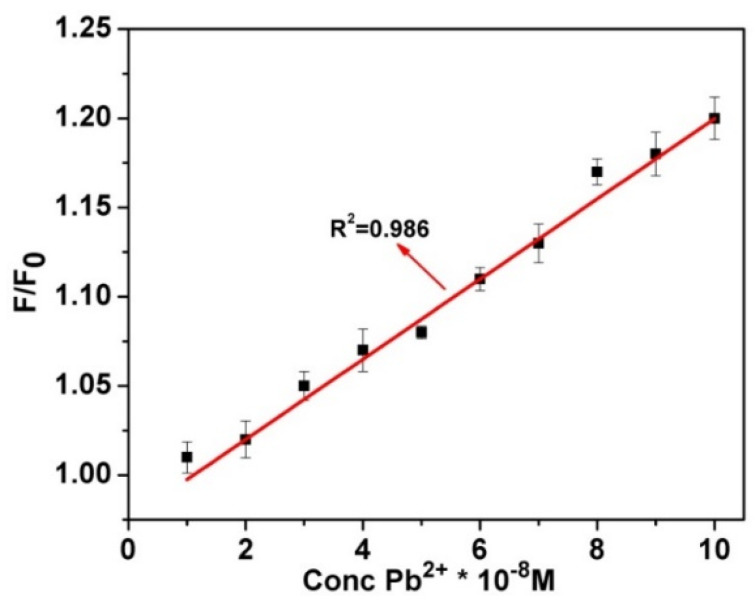
The linear variation of fluorescence intensity of WMED CQDs in triple distilled water (TDW) with increasing concentration of lead, λ_ex_ = 390 nm.

The sensitivity and linearity of the carbon quantum dots treated with ethylene diamine were investigated by varying the Pb^2+^ concentration. As shown in Figure 6, the fluorescence intensity also proportionately increased with the increase in the concentration of Pb^2+^ ions. It is evident from XPS and FTIR studies that the WMED-CQDs are well functionalized with carboxyl and amine groups. Pb^2+^ can bind to the amine group since the lone pair electrons of an amine group have a strong affinity to the outer shell of the metal ions. A strong complex formation between Pb^2+^ and functionalized WMED-CQDs leads to further stabilizing the CQDs in aqueous medium, and the agglomeration of WMED-CQDs is restricted, which blocks the non-radiative path and activates radiative decay.

The fluorescence titration experiments were performed to determine the detection limit for lead ions. The fluorescence emission spectra of WMED with different concentration of Pb^2+^ ions were measured in triplicate; and the slope and standard deviation were calculated at 469 nm i.e., at emission maxima. The detection limit was calculated using the following equation: LOD = 3.3 σ/k, where σ is the standard deviation, and k is the slope of the calibration curve between the fluorescence intensity at different concentrations of Pb^2+^ ions. The limit of detection (LOD) of WMED probe for Pb^2+^ was found to be 190 pM, which is lower than that of the maximum level (72 nM) permitted by the United States Environmental Protection Agency (EPA) [54] in drinking water.

### Detection of Pb^2+^ Ions in Polluted Water

**Figure 7:**
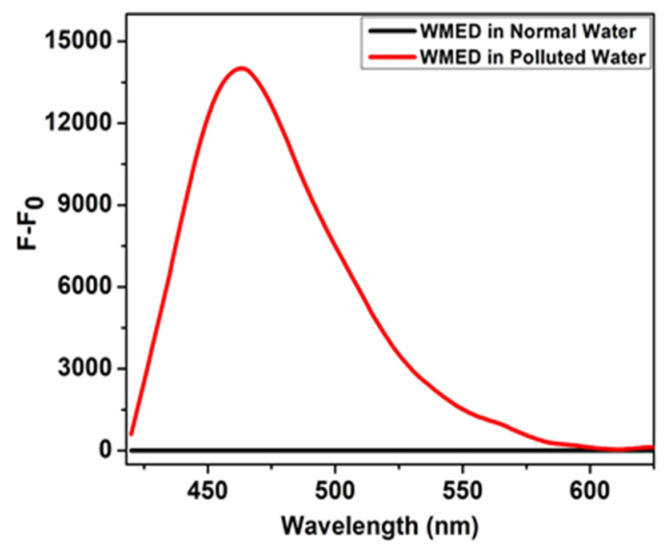
Fluorescence intensity of WMED in normal water (black line) and contaminated water having Pb^2+^ ions (Red line).

The lead-contaminated water sample was collected from the drainage pipe in the lab. The different concentration of metal ions including lead ions were thrown in the lab washbasin and contaminated water was collected. Fluorescence investigations were conducted in both normal and polluted water to assess WMED’s potential. When the fluorescence intensity of WMED in polluted water was compared with normal water, the fluorescence was enhanced in the case of contaminated water, indicating the presence of Pb^2+^ ions (Figure 7).

Meanwhile, the influence of coexisting metal ions on the selective detection of Pb^2+^ ions was also studied. The emission intensities of WMED CQDs dispersions at 470 nm after the addition of Pb^2+^ ions (10^−4^ M) alone and in the mixture of Pb^2+^ ions (10^−4^ M) and other metal ions (10^−4^ M) were measured. In the presence of other metal ions, the fluorescence enhancement produced by Pb^2+^ ions was not quenched, as shown in Supplementary Figure S7. Based on the aforementioned results, the better fluorescence sensitivity and selectivity of WMED CQDs towards Pb^2+^ ions are most likely attributed to the strong coordination contacts between the surface functional groups of WMED CQDs and the Pb^2+^ ions. 

To evaluate the cytotoxicity of the WMED CQDs, the MTT (3-(4,5-dimethylthiazolyl-2)-2,5-diphenyltetrazolium bromide) assay was performed in triplicates in a 96-well plate (Supplementary Figure S8). The cells were trypsinised and counted through a haemocytometer. In a 96-well plate, 8000 cells per well were seeded and incubated overnight in a CO_2_ incubator and then treated with the CQDs on the next day at different concentrations ranging from 0.02 to 2 mg/mL and incubated further for 24 h in a CO_2_ incubator. Post incubation, 22 μL of 5 mg mL^−1^ MTT was added to each well to get the final concentration of 0.5 mg mL^−1^ MTT reagent. These cells were further incubated for 4 h at 37 °C in the CO_2_ incubator. The media were carefully removed, and the reaction was quenched using dimethyl sulfoxide (DMSO) (200 μL). These plates were covered and agitated for 15 min on an orbital shaker; then, the absorbance was obtained at 570 nm. The Optical Density (O.D.) of the MTT-containing wells were analyzed to calculate the percent viability of the cells in the presence of different concentrations of WMED to assess their toxic effect. To determine the percent viability of the cells, the O.D. for untreated, i.e., 0 mg/mL of the CQDs was considered 100% viability and the respective values for other WMED concentrations were calculated.

**Figure 8:**
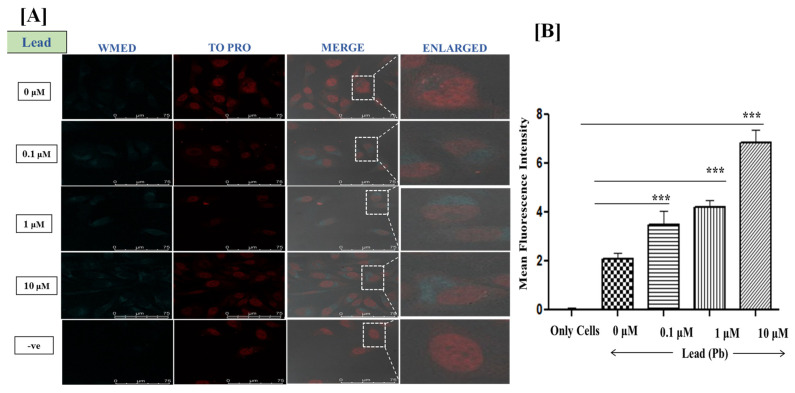
(**A**) Confocal fluorescence microscopy images at 100× Magnification; Live HeLa cells were incubated with Lead ions at 0.1 μM, 1 μM, and 10 μM concentrations for 1.5 h. Staining of Live HeLa cells with 0.128 mg/mL WMED, images recorded for WMED at λ_ex_ 405 nm/λ_em_ 450–550 nm. The Nucleus is stained with TO-PRO nuclear dye λ_ex_ 641 nm/λ_em_ 661 nm; (**B**) quantification of lead ions using ImageJ software version 1.46r, *** *p* < 0.001.

When live HeLa cells were incubated with Pb^2+^ (lead) alone, no fluorescence emission was detected through confocal laser scanning microscopy. However, live HeLa cells were treated with only carbon quantum dots of WMED (no Pb^2+^ ions) showed weak fluorescence in the cytoplasm. To check the ability of synthesized CQDs to detect Pb^2+^, we incubated these cells with different concentrations of Pb^2+^ (0.1–10 μM) followed by incubating WMED-CQDs (100 μL of 0.06 mg/mL or 0.128 g/mL) for 1 h. It showed a relatively enhanced fluorescence in comparison with only those treated with CQDs of WMED as shown in (Figure 8, Supplementary Figure S9).

Our results demonstrated that Pb^2+^ and WMED both are permeable through the living cell membrane of Hela cells and WMED fluorescence intensity amplified gradually with the increased stimulus of Pb^2+^ ranging from 0.1 μM to 10 μM (Figure 8). These cells were also stained with nuclear stain To-Pro to assess the staining pattern of WMED. WMED staining was observed mainly in the cytoplasmic region of the HeLa cells. Furthermore, cytotoxicity of WMED was assessed in live HeLa cells through an MTT (3-(4,5-dimethylthiazol-2-yl)-2,5-diphenyl tetrazolium bromide) assay to check its biocompatibility. WMED-CQDs showed no significant toxicity in HeLa cells up to 0.2 mg/mL concentration. Hence, these biocompatible carbon quantum dots of WMED can be utilized for many biological applications.

## 4. Conclusions

In summary, we have successfully prepared functionally tailored fluorescent CQDs from watermelon juice through a simple, green and cost-effective procedure. By introducing nitrogen-containing passivating ligands such as ethanolamine (EA) and ethylenediamine (ED) in watermelon juice, the remarkable ~10-fold and ~6-fold increase in fluorescence intensities were observed in the case of WMEA-CQDs and WMED-CQDs. We demonstrated that WMED CQDs detect Pb^2+^ ions in polluted water with a remarkably good limit of detection of 190 pM. The as-synthesized CQDs with different surface functionalities such as carbonyl, hydroxyl, and carboxylic acid exhibited excitation-dependent emission, good photostability, and water miscibility. WMED CQDs in an aqueous medium have shown the potential to detect Pb^2+^ ions in live cancer cells (HeLa cells) by displaying fluorescence signals. These watermelon derived CQDs can be viewed as environmentally benign nanomaterials for detection of Pb^2+^ ions in biological and environmental samples.

## Data Availability

Supporting information provided. Additional data if required will be provided on request.

## Supplementary Materials

The following supporting information can be downloaded at: https://www.mdpi.com/article/10.3390/jimaging9010019/s1. Table S1: Carbon Quantum Dots for Pb^2+^ ions. Table S2: Absorption and Emission data of CQDs. Figure S1: UV-visible absorption, emission and Excitation dependent emission spectra of (a,b) WM CQDs (c,d) WMEA CQDs (e,f) WMED CQDs (g,h) WMSA CQDs (i,j) WMEG CQDs in aqueous medium. Figure S2: Photostability test of CQDs under continuous irradiation of the 365 nm light with different time intervals. (a) Plot of the fluorescence intensity of WMEA-CQDs at emission maximum (λ_max,em_ 465 nm) and (b) plot of the fluorescence intensity of WMED-CQDs at emission maximum (λ_max,em_ 470 nm) (performed in aqueous medium with excitation wavelength of 370 nm). Figure S3: pH dependence of fluorescence response of (a) WMEA-CQDs and (b) WMED-CQDs in various pH ranges. Figure S4: High resolution XPS spectra of the WMEA CQDs in (a) O1s region, (b) N1s region and WMED CQDs in (c) O1s region (d) N1s region. Figure S5: Metal binding properties of WMED to different metal ions. The concentration of metal ions was 1 × 10^−4^ M. Figure S6: Metal binding properties of the WMEA to different metal ions. The concentration of Pb^2+^ and other metal ions was 1 × 10^−4^ M. Figure S7: Selective fluorescence response of WMED towards Pb^2+^ ions (red bar), and interference of other metal ions with Pb^2+^ ions (blue bars) in Water. F_0_ is the emission intensity of WMED in the absence of metal ions. F is the emission intensity of WMED with various metal ions. The concentration of Pb^2+^ and other metal ions was 1 × 10^−4^ M. Figure S8: Cell viability assessment of WMED in cervical cancer cell line HeLa did not show any significant toxicity up to 0.2 mg/mL concentration of WMED. Figure S9: Staining of Live HeLa cells with 0.06 mg/mL WMED Confocal microscopy images at 100× Magnification. Live HeLa cells incubated with Lead at 0.1 μM, 1 μM, and 10 μM concentration for 30 min and then WMED for 1 h. Spectra recorded for WMED at λ_ex_ 405 nm/λ_em_ 450–550 nm. Nucleus is stained with TO-PRO nuclear dye λ_ex_ 641 nm/λ_em_ 661 nm.
