# Supplementary material for: Picomolar Detection of Lead Ions (Pb2+) by Functionally Modified Fluorescent Carbon Quantum Dots from Watermelon Juice and Their Imaging in Cancer Cells
Source: J Imaging. 2023 Jan 16;9(1):19. doi: 10.3390/jimaging9010019 (PMC9865117; doi:10.3390/jimaging9010019)
Supplement: Supplementary file 1 [file jimaging-09-00019-s001.zip › jimaging-2061100-supplementary.pdf]

Supporting Information (SI)

Table of Content

UV Visible, emission and normalised fluorescence spectra and table	S2–S3
Photostability study of both CQDs under UV light exposure	S4
pH dependent study of both CQDs	S4
High resolution XPS spectra of WMEA and WMED CQDs	S5
Metal binding properties of WMED	S5
Metal binding properties of WMEA and Interference studies of WMED	S6
MTT essay and HeLa cell imaging	S7
Comparison table with References	S8

WM (without ligand treated)

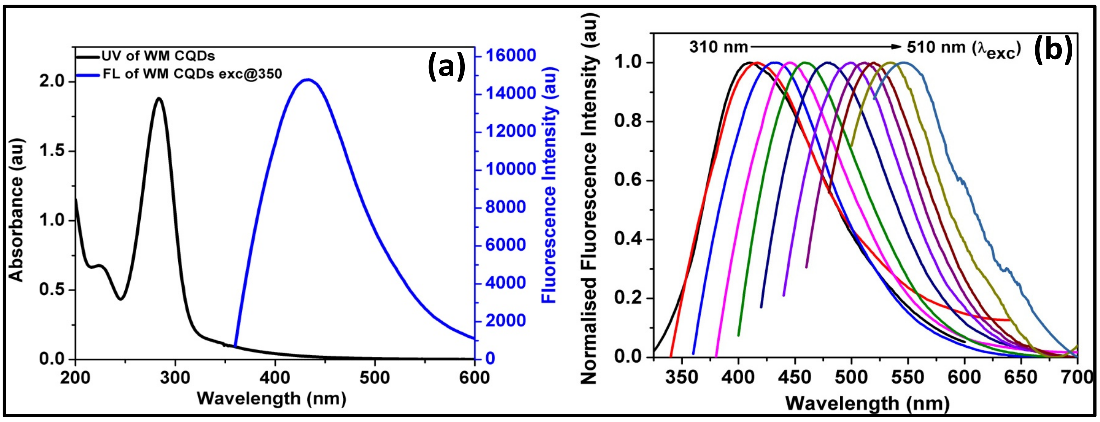

WMEA (Treated with ethanolamine)

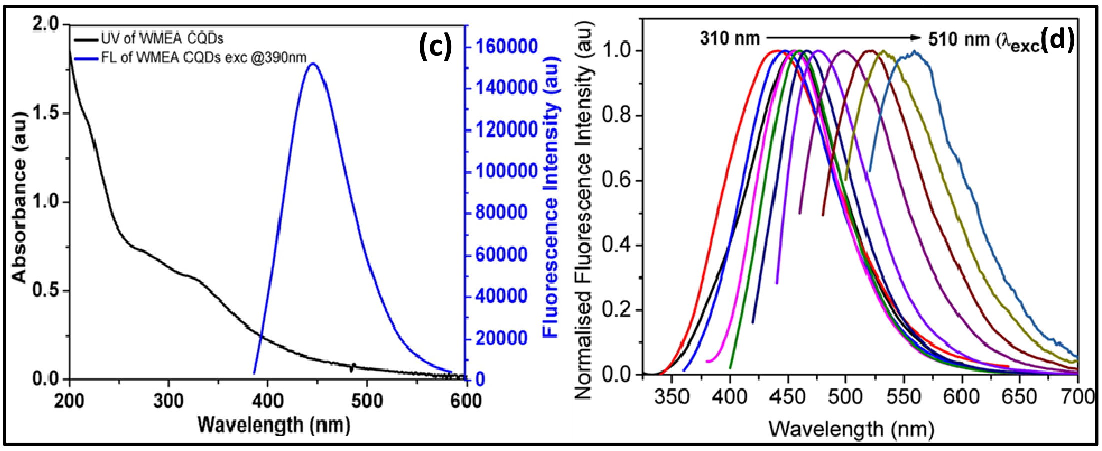

WMED (Treated with ethylene diamine)

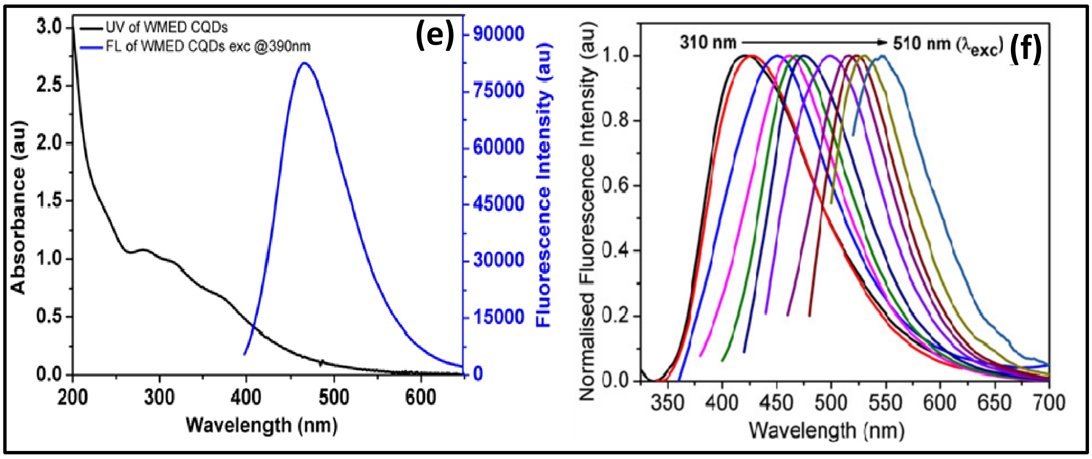

WMSA (Treated with succinic acid)

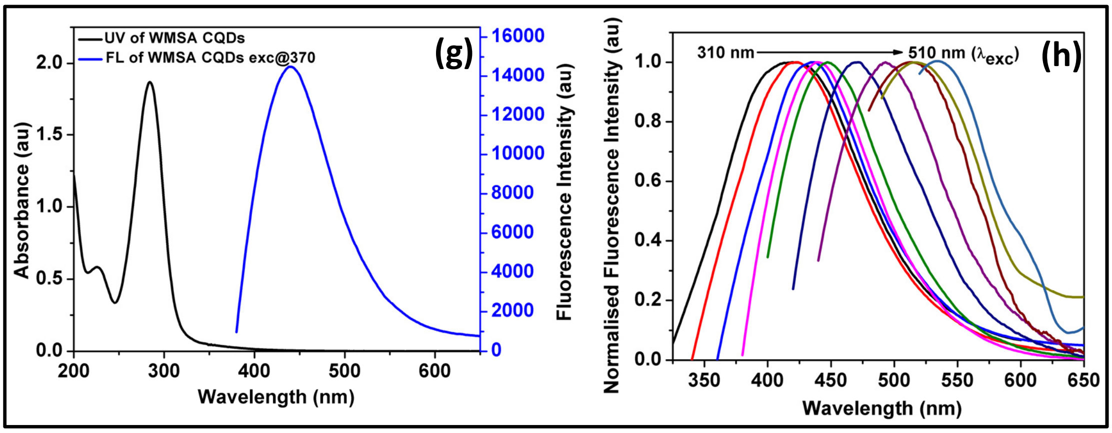

WMEG (Treated with ethylene glycol)

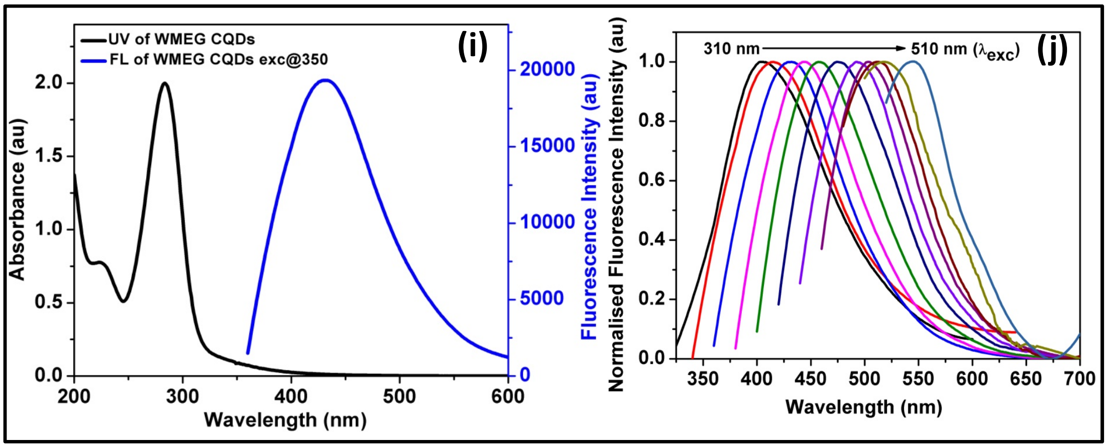

Figure S1. UV-visible absorption, emission and Excitation dependent emission spectra of (**a**,**b**) WM CQDs (**c**,**d**) WMEA CQDs (**e**,**f**) WMED CQDs (**g**,**h**) WMSA CQDs (**i**,**j**) WMEG CQDs in aqueous medium.

Table S2. Absorption and Emission values of CQDs.

S. No.	CQDs	Absorption Range (nm)	Absorbance Maxima (nm)	Excitation $_{ex}$(nm)	Emission maxima $_{em}$ (nm)
1	WM	245-335	285	350	433
2	WMEA	250-400	305	390	468
3	WMED	250-410	315	390	470
4	WMSA	245-335	283	370	441
5	WMEG	245-335	283	350	430

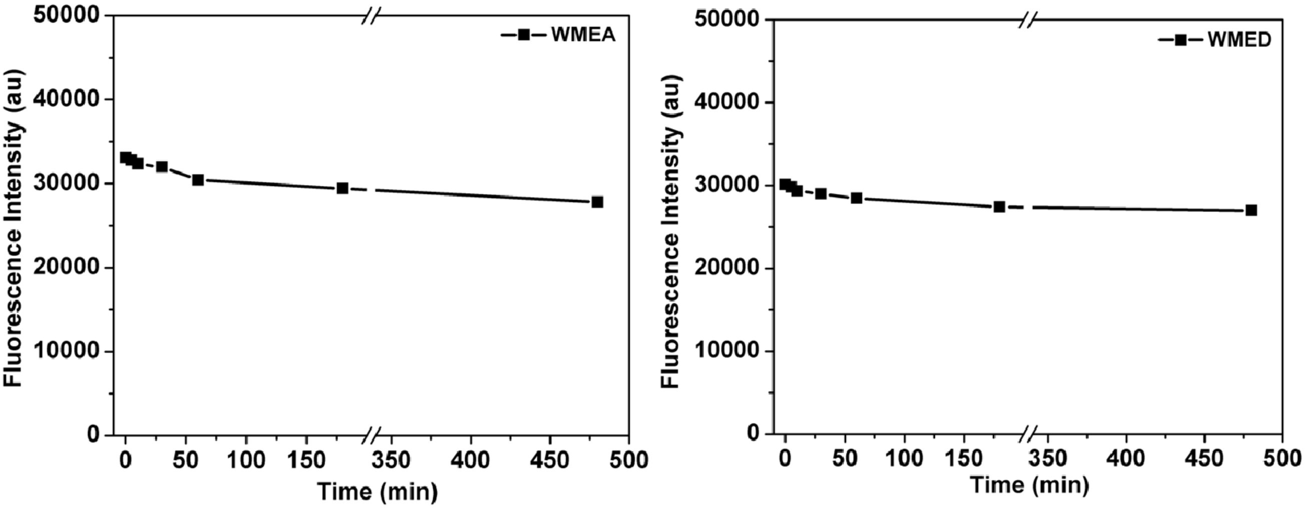

Figure S2. Photostability test of CQDs under continuous irradiation of the 365 nm light with different time intervals. (**a**) Plot of the fluorescence intensity of WMEA-CQDs at emission maximum ($_{max,em}$ 465 nm) and (**b**) Plot of the fluorescence intensity of WMED-CQDs at emission maximum ($_{max,em}$ 470 nm) (performed in aqueous medium with excitation wavelength of 370 nm).

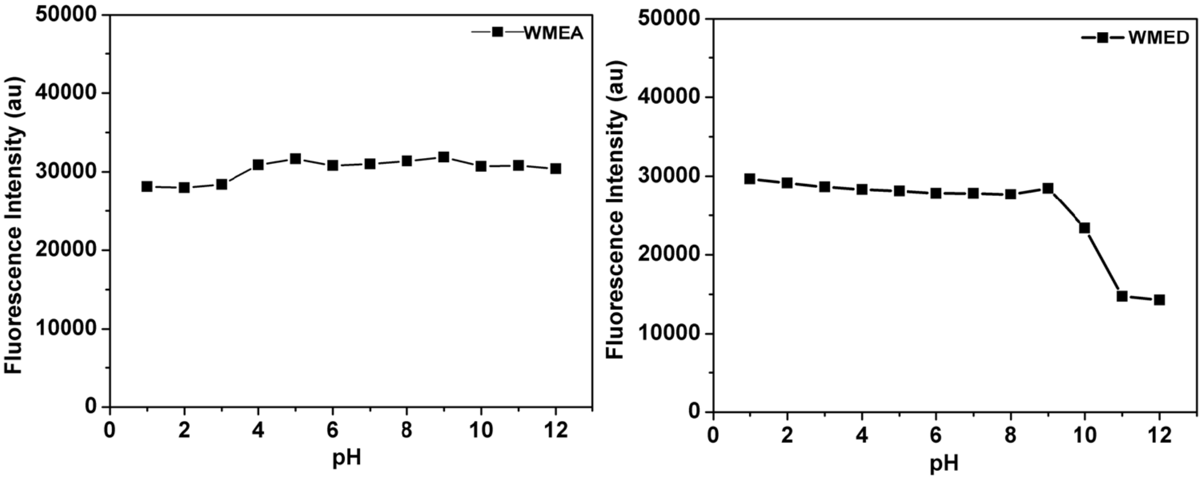

Figure S3. pH dependence of fluorescence response of (**a**) WMEA-CQDs and (**b**) WMED-CQDs in various pH ranges.

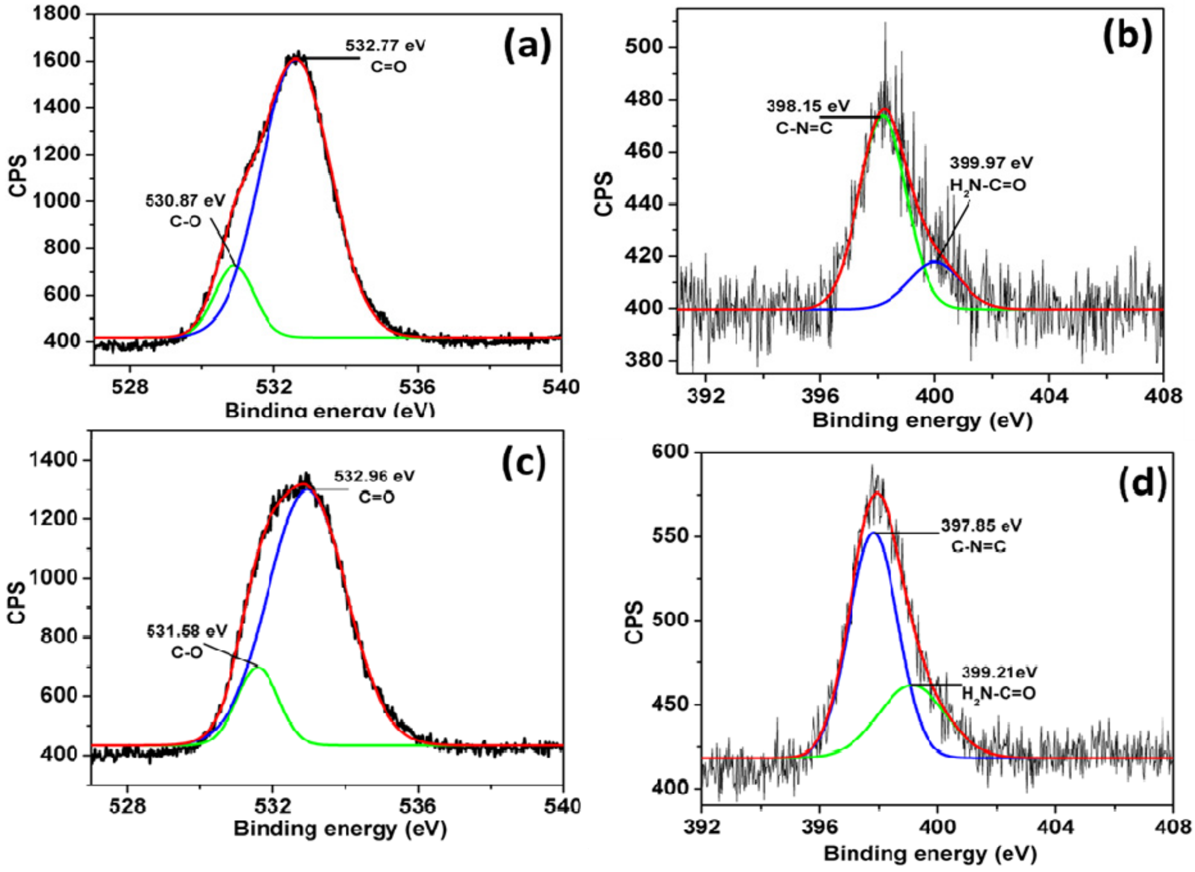

Figure S4. High resolution XPS spectra of the WMEA CQDs in (**a**) O1s region, (**b**) N1s region and WMED CQDs in (**c**) O1s region (**d**) N1s region.

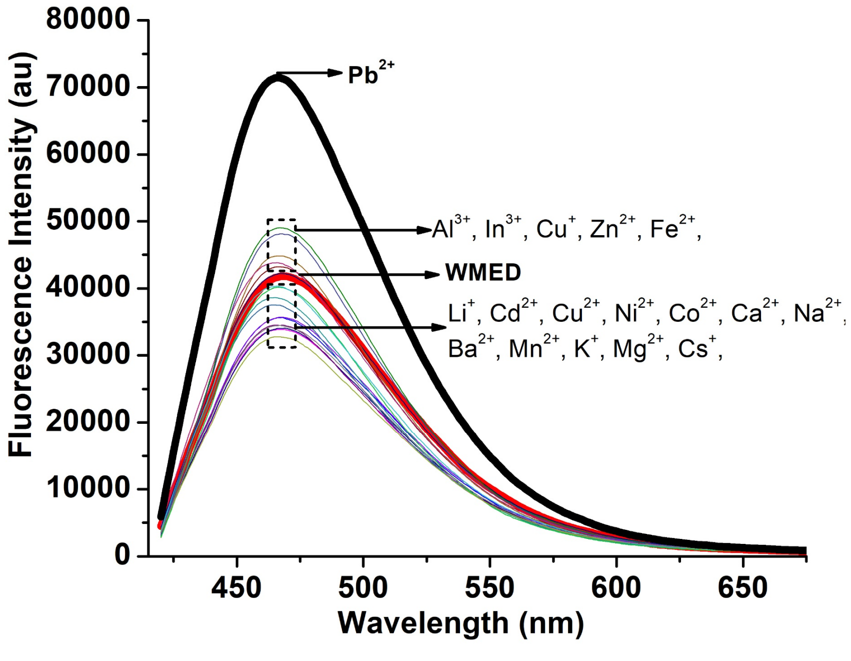

Figure S5. Metal binding properties of WMED to different metal ions. The concentration of metal ions was 1×10^{-4}M.

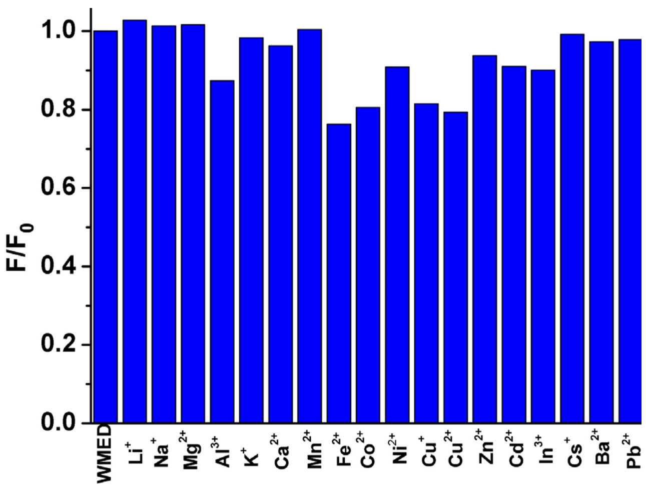

Figure S6. Metal binding properties of the WMEA to different metal ions. The concentration of Pb^{2+} and other metal ions was 1 × 10^{-4}M.

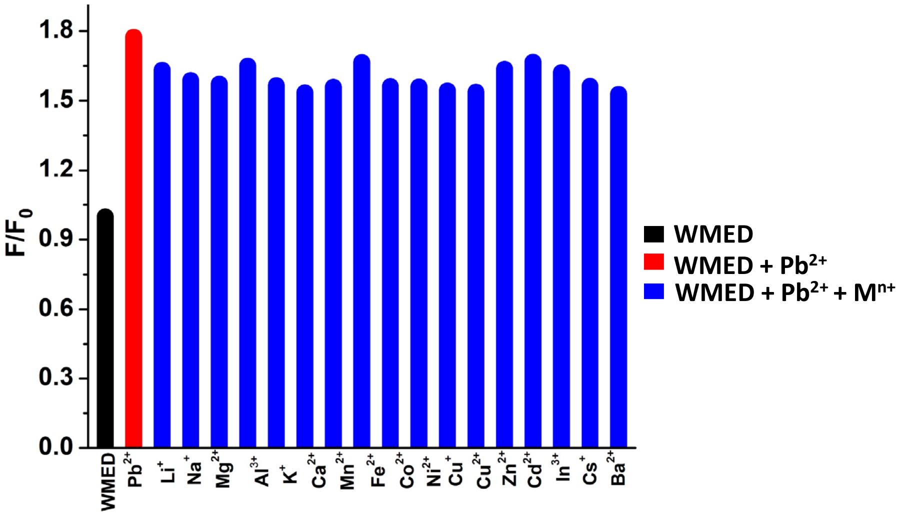

Figure S7. Selective fluorescence response of WMED towards Pb^{2+} ions (red bar), and interference of other metal ions with Pb^{2+} ions (blue bars) in Water. F_0 is the emission intensity of WMED in the absence of metal ions. F is the emission intensity of WMED with various metal ions. The concentration of Pb^{2+} and other metal ions was 1 × 10^{-4}M.

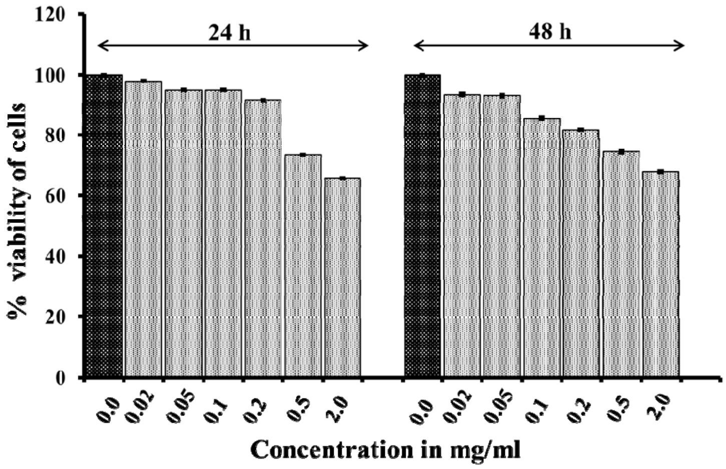

Figure S8. Cell viability assessment of WMED in cervical cancer cell line HeLa did not show any significant toxicity up to 0.2 mg/mL concentration of WMED.

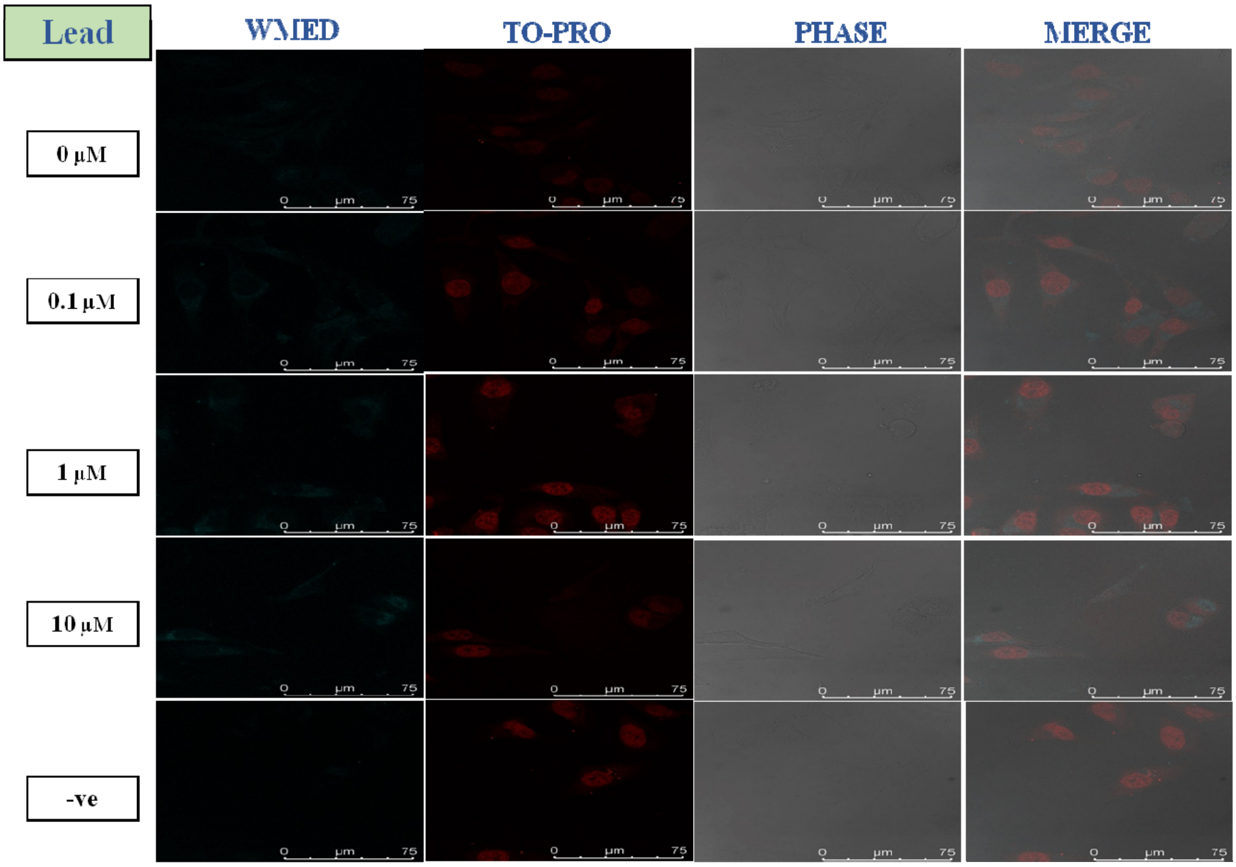

Figure S9. Staining of Live HeLa cells with 0.06mg/mL WMED Confocal microscopy images at 100X Magnification. Live HeLa cells incubated with Lead at 0.1 µM, 1 µM, and 10 µM concentration for 30 min and then WMED for 1 h. Spectra recorded for WMED at λ_{ex} 405 nm/ λ_{em} 450–550 nm. Nucleus is stained with TO-PRO nuclear dye λ_{ex} 641 nm/ λ_{em} 661 nm.

Table S1. Carbon Quantum Dots for Pb^{2+} ions.

Probe	LOD	Mechanism	References
Gallic acid–Au NPs	5.0 uM	Based on absorbance **Title** In Situ Synthesis of Metal Nanoparticles and Selective Naked-Eye Detection of Lead Ions from Aqueous Media	J. Phys. Chem.C, 2007, 111, 12839–12847.
Glutathione-Capped Quantum Dots	40nm	Quenching **Title** Ultrasensitive Pb2+ Detection by Glutathione-Capped Quantum Dots	Anal. Chem. 2007, 79, 9452–9458
Peptide–Au NPs	242 nM	Based on absorbance and no selectivity. **Title** Colorimetric Response of Peptide- Functionalized Gold Nanoparticles to Metal Ions	Small, 2008, 4, 548–551.
2-ME/S_2O_3 2_–Au NPs	0.5 nM	Quenching **Title** Colorimetric Assay for Lead Ions Based on the Leaching of Gold Nanoparticles	Anal. Chem., 2009, 81, 9433–9439.
Au NPs, DNAzyme and substrate	0.5 uM	Quenching **Title** DNAzyme-based colorimetric sensing of lead (Pb2+) using unmodified gold nanoparticle probes	Nanotechnology, 2008, 19, 095501
Au NPs, DNAzyme and substrate	3.0 nM	Absorbance **Title** Label-Free Colorimetric Detection of Lead Ions with a Nanomolar Detection Limit and Tunable Dynamic Range by using Gold Nanoparticles and DNAzyme	Adv. Mater., 2008, 20, 3263–3267.
DNA–Au NPs, DNAzyme and substrate	10 uM	**Title** Enzymatic Cleavage of Nucleic Acids on Gold Nanoparticles:A Generic Platform for Facile Colorimetric Biosensors	Small,2008, 4, 810–816.
DNA–Au NPs, DNAzyme and substrate	0.5 uM	**Title** Easy-to-use dipstick tests for detection of lead in paints using non-cross-linked gold nanoparticle–DNAzyme conjugates	Chem. Commun., 2010, 46, 1416–1418.
DNA–Au NPs, DNAzyme and substrate	0.4 uM	**Title** Accelerated Color Change of Gold Nanoparticles Assembled by DNAzymes for Simple and Fast Colorimetric Pb2+ Detection	J. Am. Chem. Soc., 2004, 126, 12298–12305.
Single-labeled hairpin	0.4 nM	Quenching **Title** Single-labeled hairpin probe for highly specific and sensitive detection of lead(II) based on the fluorescence quenching of deoxyguanosine and G-quartet	Biosensors and Bioelectronics 41 (2013) 137–142
CdSe/CdS quantum dots	0.006 nmol L^{-1}	Dithizone functionalized CdSe/CdS quantum dots as turn-on fluorescent probe for ultrasensitive detection of lead ion	Journal of Hazardous Materials 250– 251 (2013) 45–52
Graphene Quantum Dots/L-Cysteine	70 nM.	Quenching **Title** Graphene Quantum Dots/L-Cysteine Coreactant Electrochemiluminescence System and Its Application in Sensing Lead(II) Ions	ACS Appl. Mater. Interfaces 2014, 6, 1646−1651
CdSe/ZnS QDs	0.99 nmol L^{-1}	Quenching **Title** Cathodic electrochemiluminescence of a CdSe/ZnS QDs-modified glassy carbon electrode and its application in sensing of Pb^{2+}	Anal. Methods, 2015, 7, 1395–1400
CQDs from Watermelon juice using ethylenediamine as surface functionalizing agent	0.19 nM	Fluorescence enhancement **Title** Picomolar Detection of Lead Ions (Pb^{2+}) by Functionally Modified Fluorescent Carbon Quantum Dots from Watermelon Juice and their Imaging in Cancer Cells	(Present Work)
